# Anicare Book Reviews: Anicare Book Reviews: The Assessment and Treatment of Children Who Abuse Animals. By Kenneth Shapiro, Mary Lou Randour, Susan Krinsk and Joann L. Wolf. Springer: Cham, Switzerland, 2014; 124 pp; $49.99; ISBN 978-3-319-01088-5; The Identification, Assessment, and Treatment of Adults who Abuse Animals. By Kenneth Shapiro and Antonia J.Z. Henderson. Springer: Cham, Switzerland, 2016; 115 pp; $79.99; ISBN: 978-3-319-27362-4

**DOI:** 10.3390/ani6090053

**Published:** 2016-08-31

**Authors:** Catherine Tiplady

**Affiliations:** Centre of Animal Welfare and Ethics, School of Veterinary Science, University of Queensland, Gatton, QLD 4343, Australia; catherine.tiplady@uqconnect.edu.au

The connection between abuse of animals and human interpersonal violence has attracted increasing interest from researchers, professionals and the community in recent decades.

Understanding the connection between animal abuse and human interpersonal violence is vital for all who work in the human and animal welfare fields; however, many professionals (in particular, veterinarians) feel ill-equipped to recognize and support victims of abuse.

Two recent publications by Springer (*The Assessment and Treatment of Children Who Abuse Animals* and *The Identification, Assessment, and Treatment of Adults who Abuse Animals*) provide a concise and informative guide for those who work with people and animals impacted by abuse.

## The Assessment and Treatment of Children Who Abuse Animals. By Kenneth Shapiro, Mary Lou Randour, Susan Krinsk and Joann L. Wolf. Springer: Cham, Switzerland, 2014; 124 pp; $49.99; ISBN 978-3-319-01088-5

This book suggests a method of assessment and treatment for mental health professionals who work with children under 17 years of age who have perpetrated or witnessed animal abuse. The book is also intended to be of use to those in allied fields, such as domestic violence workers, childcare providers, teachers and social workers.

AniCare Child is divided into three sections—Theory, Assessment, and Treatment and is very user-friendly, with subheadings and boxed text used to highlight case studies and key points. Interventions outlined in the book can serve as the primary vehicle of treatment or as a supplement to interventions by therapists. Topics covered include the diagnostic categories associated with children who commit animal abuse, use of pet therapy for children who have abused animals, the development of empathy, effects of witnessing animal abuse and discussion of attachment theory.

Appendices are well laid out and include screening questions on animal related experiences. Supplementary electronic material includes workshop presentations and assessment and treatment material.

The focus of this book appears to be most relevant to those working with children, rather than those (e.g., shelter workers and veterinarians) who are working with the abused animals. However, despite this, the book provides much needed information for anyone who wishes to learn more about children and animal abuse.

## The Identification, Assessment, and Treatment of Adults who Abuse Animals. By Kenneth Shapiro and Antonia J.Z. Henderson. Springer: Cham, Switzerland, 2016; 115 pp; $79.99; ISBN: 978-3-319-27362-4

This book provides guidance on how to identify, assess and treat adults who have abused animals. The theoretical framework utilized covers cognitive behavioral, attachment, psychodynamic and trauma-based theories.

Topics covered are diverse and include animal abuse occurring within male or female perpetrated domestic violence, self-harm and animal abuse, the role of veterinarians and the criminal justice system and how to establish a working relationship with the client who may be compliant, resistant or defensive. The perpetrator’s gender is discussed, with higher rates of animal hoarding reported among women, higher rates of animal abuse among men and approximately equal rates between male and female genders for animal neglect.

Additional materials include an appendix of cases that illustrate client presentations and electronic supplementary material demonstrating role-played interviews and a workshop presentation.

In conclusion, the latest publications of AniCare books (for those working with children and adults who abuse animals) provide an easy to read approach and will be of great interest to all in the human and animal welfare fields. It would be ideal for the publishers to combine both AniCare books into a single volume in future editions.

